# A Novel Role of Arrhythmia-Related Gene *KCNQ1* Revealed by Multi-Omic Analysis: Theragnostic Value and Potential Mechanisms in Lung Adenocarcinoma

**DOI:** 10.3390/ijms23042279

**Published:** 2022-02-18

**Authors:** Kai-Tun Chang, Hsing-Ju Wu, Chien-Wei Liu, Chia-Ying Li, Hung-Yu Lin

**Affiliations:** 1 Department of Emergency Medicine, Show Chwan Memorial Hospital, Changhua 500, Taiwan; darren0924@yahoo.com.tw; 2 Research Assistant Center, Show Chwan Memorial Hospital, Changhua 500, Taiwan; hildawu09@gmail.com; 3 Department of Biology, National Changhua University of Education, Changhua 500, Taiwan; 4 Shueiduei Elementary School, Gukeng Township, Yulin 646, Taiwan; cwliu22@gmail.com; 5 Department of Surgery, Show Chwan Memorial Hospital, Changhua 500, Taiwan; 6 Graduate Institute of Biomedical Engineering, National Chung Hsing University, Taichung 402, Taiwan

**Keywords:** lung adenocarcinoma, KCNQ1, multi-omics, tumor immunity, cell cycle, diagnosis, prognosis, precision medicine

## Abstract

The early diagnosis, prognostic prediction, and personalized therapy of lung adenocarcinoma (LUAD) remains a challenging issue. *KCNQ1* (potassium voltage-gated channel subfamily Q Member 1) is implicated in long QT syndrome (LQTS) and cardiac arrhythmia, while its significance in LUAD remains unclear. In this study, we aimed to explore the significance of *KCNQ1* in terms of clinical value, tumor immunity, underlying mechanisms, and a precision medicine approach by means of multi-omics analysis. The association of *KCNQ1* with LUAD was first explored. Both altered variants and high expression of *KCNQ1* in a TCGA-LUAD cohort indicated a favorable outcome. *KCNQ1* levels had a negative correlation with tumor proliferation index *Ki67* levels. siRNA-knockdown of *KCNQ1* promoted the migration ability of lung cancer cells. *KCNQ1* levels were decreased in LUAD tissue compared to normal tissue. A receiver operating characteristic (ROC) curve indicated good diagnostic efficiency of *KCNQ1*. High *KCNQ1* is associated with an immunoactive profile of immune infiltration and immunomodulators and is involved in the inhibition of the cell cycle and DNA replication. Lapatinib was identified as a potent drug for LUAD in the context of low *KCNQ1*. This study unveiled the significance of *KCNQ1* in diagnosis and prognosis and provided a corresponding precision medicine strategy for LUAD.

## 1. Introduction

Lung cancer is the second most diagnosed cancer and the leading cause of global cancer-related deaths [1]. Non-small cell lung carcinoma (NSCLC) and small-cell lung carcinoma (SCLC) are the two main groups of lung cancer. NSCLC is the most common subtype, 40% of which accounts for lung adenocarcinoma (LUAD), 25% for squamous cell carcinoma (SCC), 15% for large cell carcinoma (LCC), and the remaining 20% for unspecified cancers [2]. When diagnosed, most patients are in the advanced stages [3]. Metastasis accounts for the primary cause of death. The identification of targetable alterations to, e.g., EGFR (epidermal growth factor receptor), ALK (anaplastic lymphoma kinase), PI3KCA/AKT1/mTOR (phosphatidylinositol-4,5-bisphosphate 3-kinase catalytic subunit alpha/AKT serine/threonine kinase 1/ mechanistic target of rapamycin kinase), RAS-MAPK (mitogen-activated protein kinases), RET (ret proto-oncogene), MET (MET proto-oncogene), BRAF (B-Raf proto-oncogene), and NTRK/ROS1 (Neurotrophic Receptor Tyrosine Kinase/ROS Proto-Oncogene 1), along with immunotherapy, has evolved the treatment paradigm in patients with advanced disease [4,5,6]. Despite these new therapeutic options, there continue to be significant challenges, as resistance development and disease progression occurs in most of these patients [7].

The *KCNQ1* (potassium voltage-gated channel subfamily Q Member 1) gene is located on chromosome 11 and consists of 17 exons of different lengths. *KCNQ1* encodes for the pore-forming alpha subunit of a voltage-gated potassium channel that enables a K^+^ current after electrical depolarization of the cell membrane. *KCNQ1* is predominantly expressed in the cells of the lungs, heart, inner ear, stomach, intestine and pancreas, in which *KCNQ1* expression is critical for ion homeostasis [8,9]. In human lung cancer cells, *KCNQ1* acts to regulate basal cAMP-stimulated Cl^−^ secretion through the cystic fibrosis transmembrane conductance regulator (CFTR) [8,10,11,12]. In addition, mutations in cardiac KCNQ1 channels account for the most common congenital defects that cause long QT syndrome (LQTS) [13,14,15], which is a heart disorder resulting in cardiac arrhythmias and about 3000 sudden deaths [16,17].

An increasing volume of studies have documented the involvement of *KCNQ1* in human cancers, including colorectal cancer, hepatocellular carcinoma, esophageal cancer, and renal cell carcinoma [18,19,20]. However, the clinical significance and biological role of *KCNQ1* in LUAD remains unclear. The aim of this study was to explore the theragnostic significance of *KCNQ1* by multi-omic analysis. Firstly, we analyzed the association between *KCNQ1* and cancers and confirmed its tumor suppression role in LUAD. Secondly, we investigated the diagnostic and prognostic vale of *KCNQ1*. Thirdly, its potential role in tumor immunity and underlying molecular basis were examined. Finally, we employed the genomics of a Drug Sensitivity in Cancer (GDSC)-based analysis to identify lapatinib as a potential candidate for the treatment of LUAD in the context of low *KCNQ1* expression.

## 2. Results

### 2.1. KCNQ1 as an Independent Risk Factor and a Tumor Suppressor in LUAD

**Figure 1:**
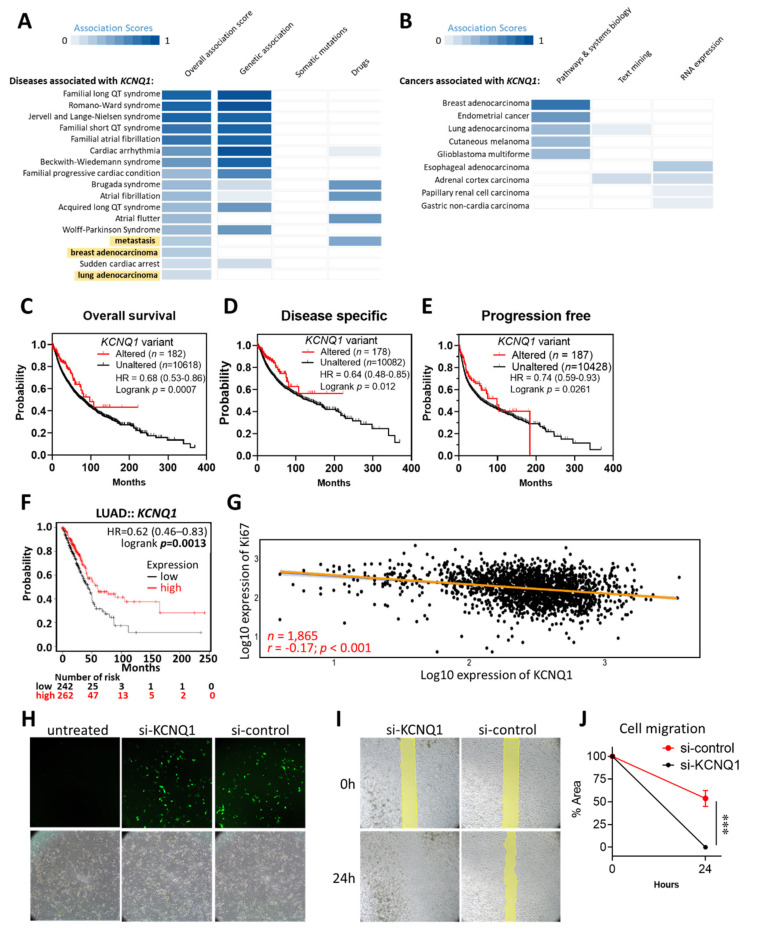
Altered variants and high expression of kcnq1 predict a favorable outcome in LUAD. Associations between *KCNQ1* and human diseases (**A**) and caners (**B**). The association scores are estimated by calculating a harmonic sum using the weighted vector of data source association scores for all data sources, summarizing all the aggregated evidence for an association. Kaplan–Meier analysis of the overall survival (**C**), disease-specific survival (**D**), and progression-free survival (**E**) in LUAD patients based on altered/unaltered *KCNQ1* variant. Kaplan–Meier analysis of the overall survival in LUAD patients based on high/low *KCNQ1* expression (**F**). Pearson correlation coefficient of the expression of *KCNQ1* with *Ki67* in LUAD tumor tissues (*n* = 1865) (**G**). HR, hazard ratio. Representative image of A549 lung cancer cells left untreated or undergoing FAM-labeled siRNAs (si-KCNQ1 and si-control) photographed under a fluorescence microscope 16 h after transfection (**H**). Representative image at 0 h and 24 h of wound healing assay (**I**). Quantification histogram (mean ± SD) of three independent experiments representing the area of the scratched ditch determined by the migrated cells (**J**). *** *p* < 0.001 between indicated groups.

We firstly illustrated the functional involvement of *KCNQ1* in human diseases using the Open Target Platform, which integrates evidence from genetics, genomics, transcriptomics, drugs, animal models, and scientific literature relevant to the association between targets and diseases [21,22]. We noted that *KCNQ1* was implicated in disease items “metastasis”, “breast adenocarcinoma”, and “lung adenocarcinoma”, aside from its previously recognized role in LQTS (Figure 1A). In light of this, we further examined the associations between *KCNQ1* and tumors and revealed some cancer types, including breast adenocarcinoma, endometrial cancer, lung adenocarcinoma, cutaneous melanoma, and glioblastoma multiforme (Figure 1B). As *KCNQ1* variants have been shown to have an association with pancreatic cancer risk [23], we then examined its impact on the progression of LUAD by accessing the cBioPortal web server. As shown in Figure 1C–E, cancer patients with altered *KCNQ1* variants showed significant longer overall survival, disease specific survival, and progression free survival. Notably, we found that high expression of *KCNQ1* indicated longer overall survival time in LUAD (Figure 1F). We then asked whether *KCNQ1* is associated with tumor proliferation by analyzing Pearson correlation coefficients of *KCNQ1* with *Ki67*. As shown in Figure 1G, *KCNQ1* was positively correlated with *Ki67* in 1865 LUAD samples (*r* = −0.17, *p* < 0.0001) (Figure 1G). We further used a siRNA-based approach to confirm the role of *KCNQ1* in an A549 human lung adenocarcinoma epithelial cell line. The transfection efficiency of FAM-labeled siRNAs was visualized under a fluorescence microscope (Figure 1H). Then, the wound healing assay showed that cells treated with si-KCNQ1 had superior migration abilities compared to cells with si-control (Figure 1I,J), thereby verifying the anti-tumor effect of KCNQ1.

### 2.2. Mutation Landscape of KCNQ1

**Figure 2:**
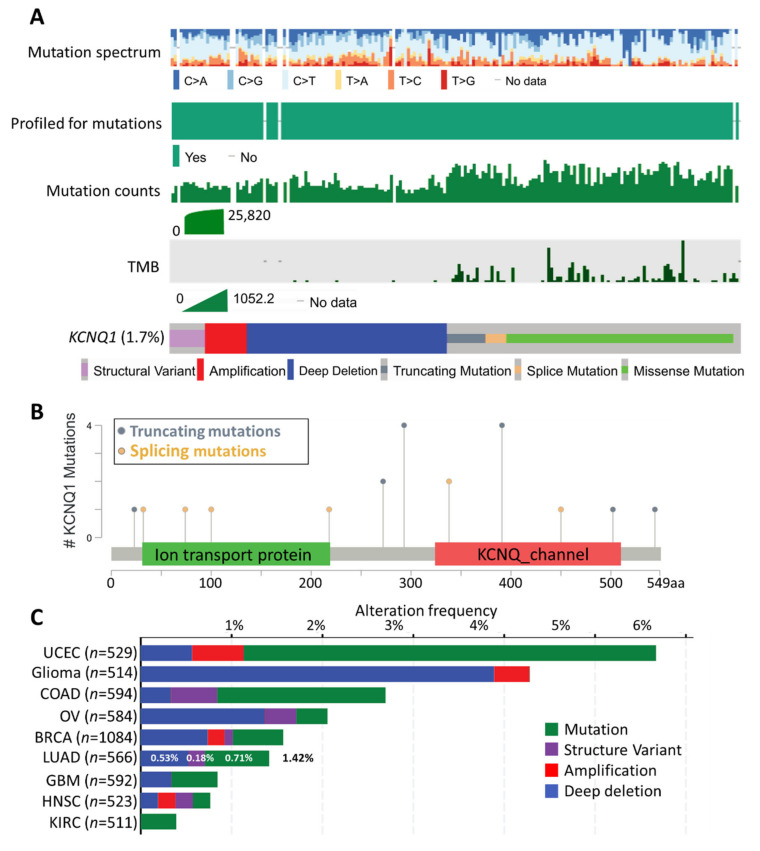
Mutation landscapes of *KCNQ1*. The mutation spectrum, profiled for mutations, mutation counts, tumor mutational burden (TMB), and genetic alterations of *KCNQ1* across all cancer patients (*n* = 10,395) (**A**). Schematic illustration number and the distribution of the mutations spanning the 549 amino-acid KCNQ1 sequence (**B**). The alteration frequency of *KCNQ1* in various cancer types (**C**). BRCA, Breast invasive carcinoma; COAD, Colon adenocarcinoma; GBM, Glioblastoma multiforme; HNSC, Head and neck squamous cell carcinoma; KIRC, Kidney renal clear cell carcinoma; LUAD, Lung adenocarcinoma; OV, Ovarian serous cystadenocarcinoma; UCEC, Uterine corpus endometrial carcinoma.

The mutational landscape of *KCNQ1* in 10,395 patients was accessed by cBioPortal analysis. As shown in Figure 2A, the mutation spectra/count of *KCNQ1* was not in synchronicity with the corresponding tumor mutational burden (TMB). The genetic alteration frequency of *KCNQ1* was 1.7%, composed of structural variants, amplification, deep deletion, truncated mutation, splicing mutation, and missense mutation. Interestingly, we observed that the occurrence of TMB was primarily accompanied by the truncated mutation, splicing mutation, and missense mutation of *KCNQ1* (Figure 2A). The number and the distribution of truncating mutations and splicing mutations across the 549 amino acid KCNQ1 is illustrated in Figure 2B. The *KCNQ1* alteration frequency in 566 LUAD cases was 1.42% (8 cases), which consists of 0.53% (3 cases) deep deletions, 0.18% (1 case) structural variants, and 0.71% (4 cases) mutations (Figure 2C).

### 2.3. Differential Expression Profile and Diagnostic Efficacy of KCNQ1

**Figure 3:**
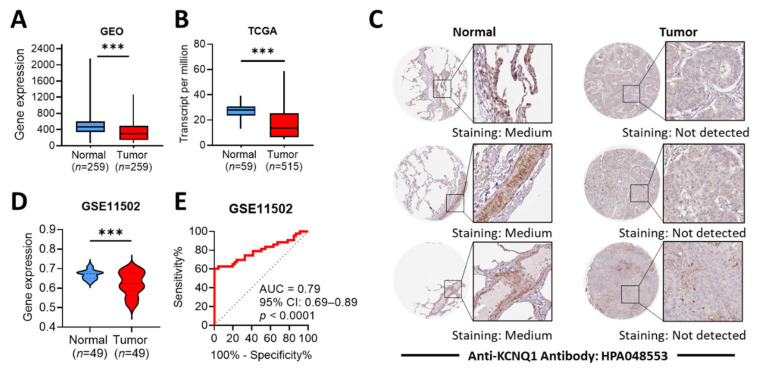
Differential expression profile and diagnostic efficacy of *KCNQ1*. The gene expression levels of *KCNQ1* in normal tissue and LUAD tumors retrieved from the GEO database (**A**) and TCGA datasets (**B**). Immunohistochemistry staining for the detection of KCNQ1 protein expression levels in normal and LUAD tissues (**C**). Validation test on the *KCNQ1* expression in normal tissue and LUAD tumors using GSE11502 datasets (**D**). Receiver operating characteristic (ROC) curves for normal and LUAD cohorts based on *KCNQ1* levels from the GSE11502 dataset (**E**). *** *p* < 0.001 between indicated groups.

The gene chip-based Gene Expression Omnibus (GEO) datasets revealed that LUAD tumors had lower *KCNQ1* expression levels than normal tissues (Figure 3A). The RNA seq-based The Cancer Genome Atlas (TCGA) datasets showed a similar pattern (Figure 3B). In addition, immunohistochemistry (IHC) staining confirmed that LUAD tumors presented lower KCNQ1 protein expression levels than normal (Figure 3C). We further verified the aforementioned observation by means of GSE11502 datasets. Similarly, *KCNQ1* expression levels were decreased in LUAD tumors compared to normal tissue (Figure 3D). To determine the diagnostic efficiency for LUAD, we constructed a receiver operating characteristic (ROC) curve and noted that *KCNQ1* expression levels presented good diagnostic accuracy for the discrimination of disease, with an area under the curve (AUC) of 0.79 (95% CI: 0.69–0.89, *p* < 0.0001) (Figure 3E).

### 2.4. Prognostic Value of KCNQ1

**Figure 4:**
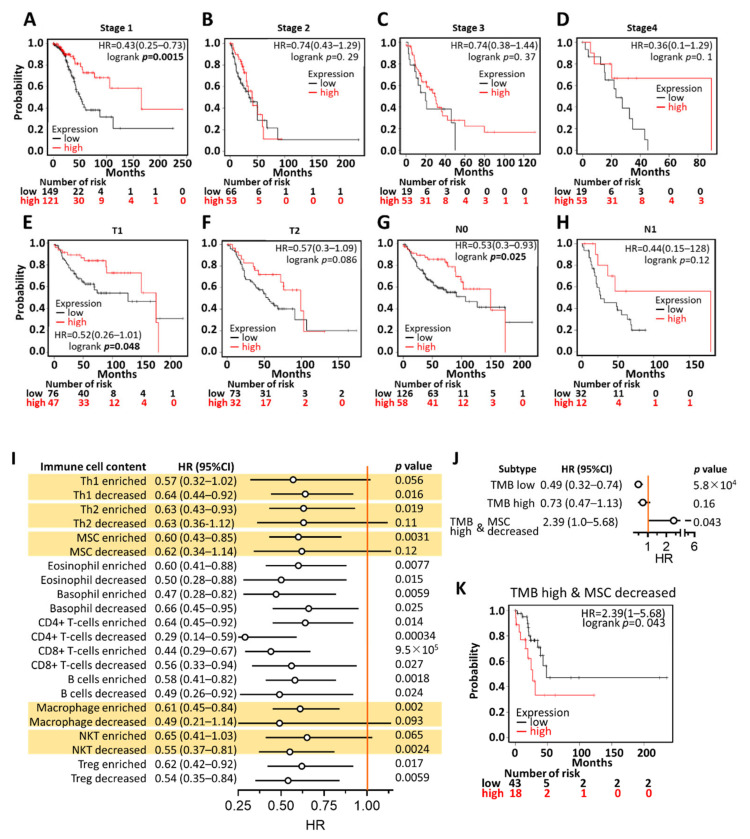
Prognostic value of *KCNQ1* in LUAD patients. Kaplan–Meier survival analysis representing the probability of overall survival based on low/high expression of *KCNQ1* in the scenario of stage 1 (**A**), stage 2 (**B**), stage 3 (**C**), stage 4 (**D**), AJCC stage T1 (**E**), AJCC stage T2 (**F**), AJCC stage N0 (**G**), and AJCC stage N1 (**H**). A forest plot summarizing the hazard ratio (HR), the 95% confidence interval (95% CI), and the logrank *p*-value of LUAD *KCNQ1* expression in the context of various immune cell contents (**I**), of high/low tumor mutation burden (TMB), or of high TMB combined with decreased MSC (**J**). Kaplan–Meier analysis based on low/high *KCNQ1* expression in the presence of high TMB and decreased MSC (**K**).

To gain more prognostic insight, the correlation between *KCNQ1* expression and corresponding clinical follow-up information was analyzed by means of Kaplan–Meier curves and the log-rank test. High *KCNQ1* expression was found to be significantly associated with increased overall survival time in LUAD patients with stage 1 cancer, but not stages 2, 3, and 4 (Figure 4A–D). In addition, this tendency was observed in patients with AJCC stage T1 and N0, but not in AJCC stage T2 and N1 (Figure 4E–H). This indicates *KCNQ1′s* prognostic value preferentially in the early stage of LUAD.

We further scrutinized the prognostic significance of *KCNQ1* expression levels in various immune cell contents. As shown in the forest plot in Figure 4I, high *KCNQ1* expression did not statistically indicate longer overall survival time compared to low *KCNQ1* expression when LUAD tumors harbored enriched type 1 helper T cells (Th1) (*p* = 0.056), decreased type 2 helper T cells (Th2) (*p* = 0.11), decreased mesenchymal stem cells (MSC) (*p* = 0.12), decreased macrophages (*p* = 0.093), and enriched natural killer T cells (NKT). Moreover, we noted that *KCNQ1* expression exhibited no prognostic significance in tumors with high TMB. Interestingly, high *KCNQ1* expression turned out to indicate shorter overall survival time when LUAD simultaneously harbored high TMB and decreased MSC (Figure 4J,K). Together, high *KCNQ1* expression levels feature prognostic value by predicting a favorable outcome in LUAD patients, while the contents of Th1, Th2, MSC, macrophages, NKT, and TMB can counteract the significance.

### 2.5. Relationship of KCNQ1 Expression with Immune Infiltration and Immunomodulators

**Figure 5:**
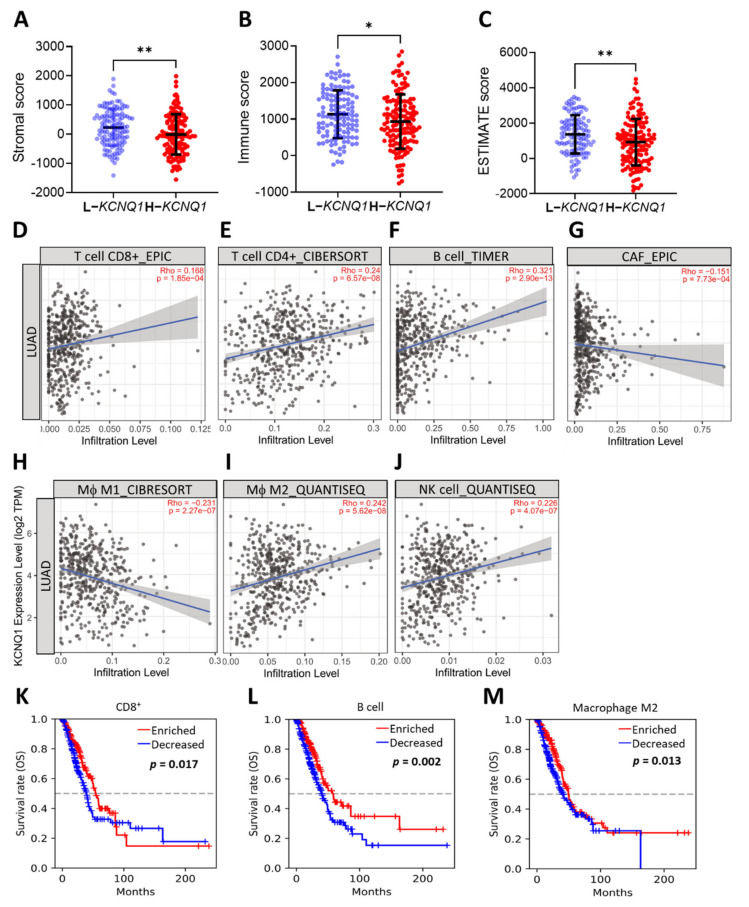
*KCNQ1* is closely related to immune infiltration. ESTIMATE analysis of stromal score (**A**), immune score (**B**), and ESTIMATE score (**C**). * *p* < 0.05, ** *p* < 0.01 between indicated groups. TIMER analysis of Spearman correlation between the *KCNQ1* expression and the infiltration of CD8+ T cells (**D**), CD4+ T cells (**E**), B cells (**F**), cancer associated fibroblasts (CAF) (**G**), (Mϕ) M1 macrophages (**H**), (Mϕ) M2 macrophages (**I**), natural killer (NK) cells (**J**). Kaplan–Meier analysis based on enriched/decreased content of CD8+ T cells (**K**), B cells (**L**), and M2 macrophages (**M**) in the LUAD cohort.

**Figure 6:**
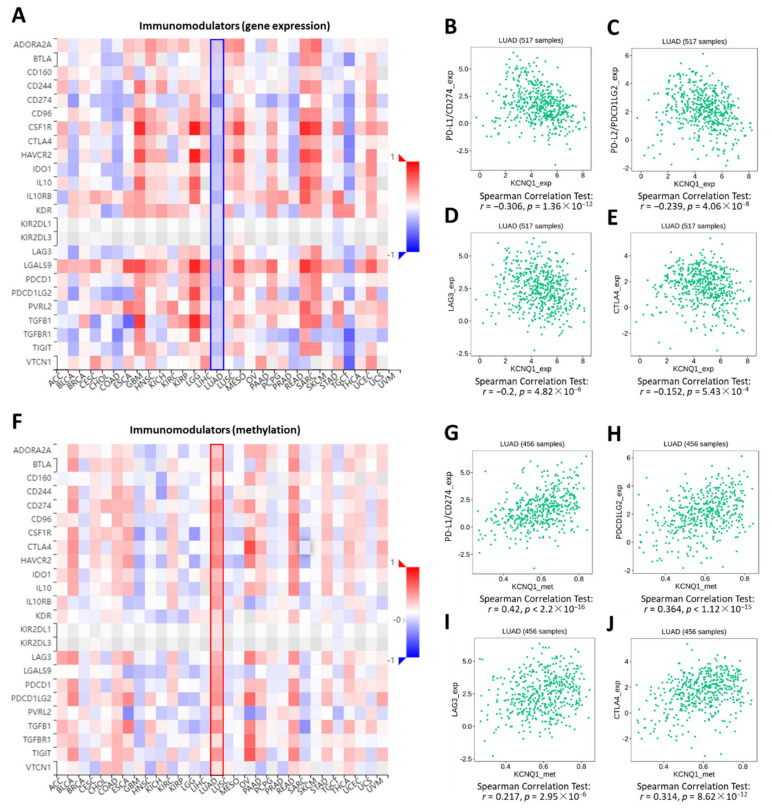
*KCNQ1* is closely related to the expression levels of immunomodulators. Correlation analysis between the expression of *KCNQ1* and 24 immunoinhibitory genes across human cancers (**A**). Spearman’s correlation test of *KCNQ1* with PD-L1/CD274 (**B**), PD-L2/PDCD1LG2 (**C**), LAG3 (**D**), and CTLA4 (**E**) in LUAD samples (*n* = 517). Correlation analysis between the expression of *KCNQ1* and methylation levels of 24 immunoinhibitory genes across human cancers (**F**). Spearman’s correlation test of *KCNQ1* with methylation levels of PD-L1/CD274 (**G**), PD-L2/PDCD1LG2 (**H**), LAG3 (**I**), and CTLA4 (**J**) in LUAD samples (*n* = 456).

Estimation of STromal and Immune cells in MAlignant Tumor tissues using Expression data (ESTIMATE) analysis was employed to determine the stromal score, immune score, and ESTIMATE score between a high *KCNQ1* group and a low *KCNQ1* group in a TCGA-LUAD cohort [24]. The high *KCNQ1* group a had lower stromal score (*p* < 0.01), immune score (*p* < 0.05), and ESTIMATE score (*p* < 0.01) (Figure 5A–C and Supplementary File S1). Based on the fact that the ESTIMATE score is inversely correlated with tumor purity [24], this result indicates that high *KCNQ1* expression is associated with lower tumor purity, thereby influencing the immune status of the tumor microenvironment. Next, TIMER analysis was used to examine the relationship between the *KCNQ1* expression and immune infiltration in LUAD. *KCNQ1* expression was significantly correlated with CD8+ T cells (*r* = 0.169, *p* = 1.85 × 10^−4^), CD4+ T cells (*r* = 0.24, *p* = 6.57 × 10^−8^), B cells (*r* = 0.321, *p* = 2.90 × 10^−13^), cancer-associated fibroblasts (*r* = −0.151, *p* = 7.73 × 10^−4^), M1 macrophages (*r* = −0.231, *p* = 2.27 × 10^−7^), M2 macrophages (*r* = 0.242, *p* = 5.62 × 10^−8^), and NK cells (*r* = 0.226, *p* = 4.07 × 10^−7^) (Figure 5D–J). We further verified the association between particular immune cell contents and overall survival in LUAD patients by Q-omics analysis. Enriched CD8+ T cells, B cells, and M2 macrophages were associated with a favorable outcome (Figure 5K–M). Specifically, TISIDB analysis revealed that the Spearman correlation coefficients for the majority of the immunomodulators were less than zero, indicating that *KCNQ1* expression was negatively correlated with the levels of immunomodulators (Figure 6A), such as PD-L1 (Figure 6B), PD-L2 (Figure 6C), LAG3 (Figure 6D), and CTLA4 (Figure 6E). In addition, we noted that *KCNQ1* expression was positively correlated with methylation levels of the immunomodulators (Figure 6F–J), suggesting that epigenetic modification may be implicated in the alteration of immune profiling. Overall, the negative correlation of *KCNQ1* with immunomodulator profiling and its simultaneous positive correlation with methylation correlation indicates a close relationship between *KCNQ1* and immunomodulators.

### 2.6. Potential Mechanism of KCNQ1 Lies in Inhibition of Cell Cycle and DNA Replication

**Figure 7:**
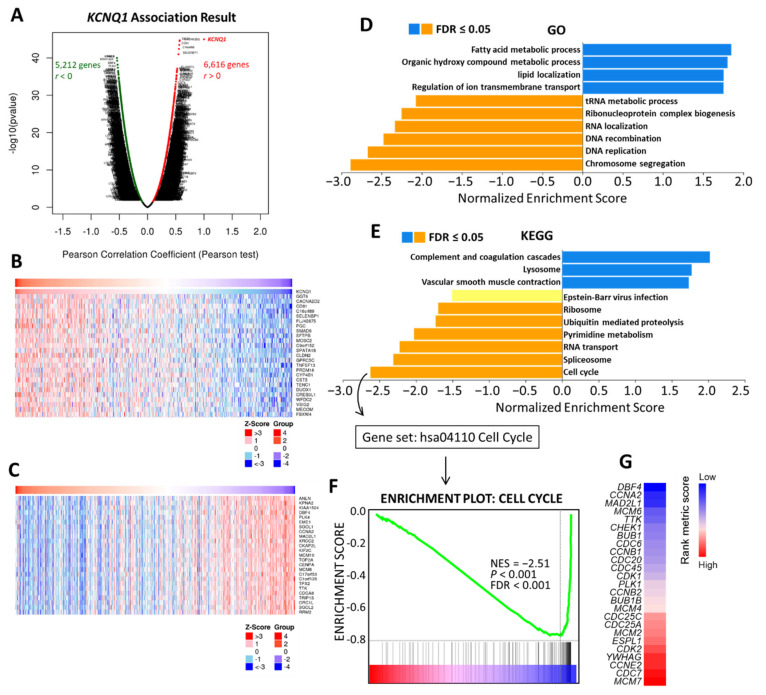
Co-expression genes of *KCNQ1* and analysis of functional enrichment. Genes significantly correlated with *KCNQ1* identified by Pearson correlation analysis in the LUAD cohort (**A**). Heatmaps illustrating the top 25 genes positively (**B**) and negatively correlated with *KCNQ1* in LUAD cohort (**C**). GO process term (**D**) and KEGG pathways (**E**) significantly enriched in *KCNQ1*-coexpressed genes in the LUAD cohort. Enrichment plots of the cell cycle functional gene set. NES, normalized enrichment score; FDR, false discovery rate (**F**). Heatmap showing the rank metric score of core enrichment genes for the gene class of the cell cycle (**G**). The genes then underwent Kaplan–Meier analysis to confirm the clinical relevance.

**Figure 8:**
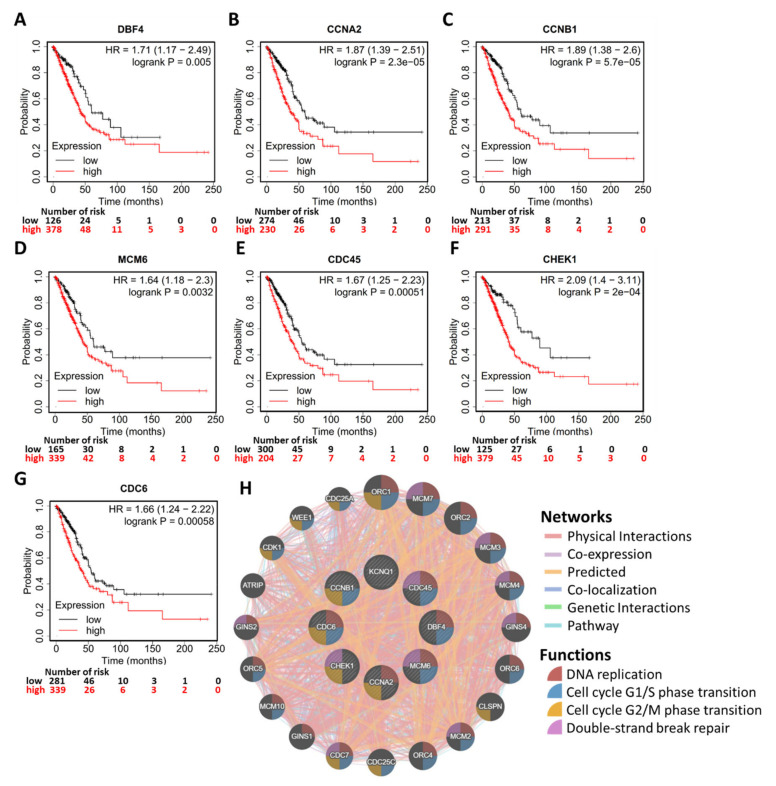
Validation of the clinical relevance of KCNQ1-coexpressed genes and the PPI. Kaplan–Meier survival analysis representing the probability of overall survival based on low/high expression of DBF4 zinc finger (DBF4) (**A**), cyclin A2 (CCNA2) (**B**), cyclin B1 (CCNB1) (**C**), minichromosome maintenance complex component 6 (MCM6) (**D**), cell division cycle 45 (CDC45) (**E**), checkpoint kinase 1 (CHEK1) (**F**), and cell division cycle 6 (CDC6) (**G**). PPI of KCNQ1 and the seven coexpression genes demonstrating that enriched functions mainly participate in DNA replication, cell cycle G1/S phase transition, cell cycle G2/M phase transition, and double-strand break repair (**H**).

To gain insight into the biological roles of *KCNQ1*, we subsequently exploited the functional modules in Linkedomics [25] to examine co-expression genes and conduct a functional enrichment analysis for the TGCA-LUAD cohort. A total of 6616 genes showed significant positive correlation with *KCNQ1*, while 5212 genes had significant negative correlations (Supplementary File S2 and Figure 7A). Heat maps revealed the top 25 genes with the most significant positive and negative correlations with *KCNQ1*, respectively (Figure 7B,C). As shown in Figure 7D, annotations of significantly enriched gene ontology (GO) terms by gene set enrichment analysis (GSEA) demonstrated that genes co-expressed with *KCNQ1* were involved in the activation of the following terms: the fatty acid metabolic process, the organic hydroxy compound metabolic process, lipid localization, and regulation of ion transmembrane transport. On the other hand, activities related to the following terms were inhibited: the tRNA metabolic process, ribonucleoprotein complex biogenesis, RNA localization, DNA recombination, DNA replication, and chromosome segregation (Figure 7D). In terms of the Kyoto Encyclopedia of Genes and Genomes (KEGG) pathway analysis, enriched genes were implicated in activating the following pathways: complement and coagulation cascades, lysosome pathways, and vascular smooth muscle contraction (Figure 7E). In contrast, inhibited pathways include the ribosome pathway, ubiquitin-mediated proteolysis, pyrimidine metabolism, RNA transport, spliceosome, and the cell cycle (Figure 7E). We noted that the resultant inhibited biological functions and pathways indicate that *KCNQ1*-coexpressed genes may be implicated in perturbations to proliferation and the cell cycle. In view of this, we further examined the cell cycle functional gene set (Figure 7F) (Supplementary File S3) and identified the core enrichment genes (Figure 7G). Then the core enrichment genes underwent Kaplan–Meier analysis. We identified that the high expression of seven genes was associated with longer overall survival time in LUAD patients, including DBF4 (DBF4 zinc finger) (Figure 8A), CCNA2 (cyclin A2) (Figure 8B), CCNB1 (cyclin B1) (Figure 8C), MCM6 (minichromosome maintenance complex component 6) (Figure 8D), CDC45 (cell division cycle 45) (Figure 8E), CHEK1 (checkpoint kinase 1) (Figure 8F), and CDC6 (cell division cycle 6) (Figure 8G). Using the GeneMANIA algorithm, the PPI network was constructed and exhibited that the enriched functions mainly participate in DNA replication, cell cycle G1/S phase transition, cell cycle G2/M phase transition, and double-strand break repair (Figure 8H). The PPI results therefore are in tandem with the results explored at the transcriptomic level. These findings suggest that *KCNQ1* may act to inhibit the cell cycle and DNA replication in LUAD.

### 2.7. Generalization Values of KCNQ1 in Pan-Cancers

**Figure 9:**
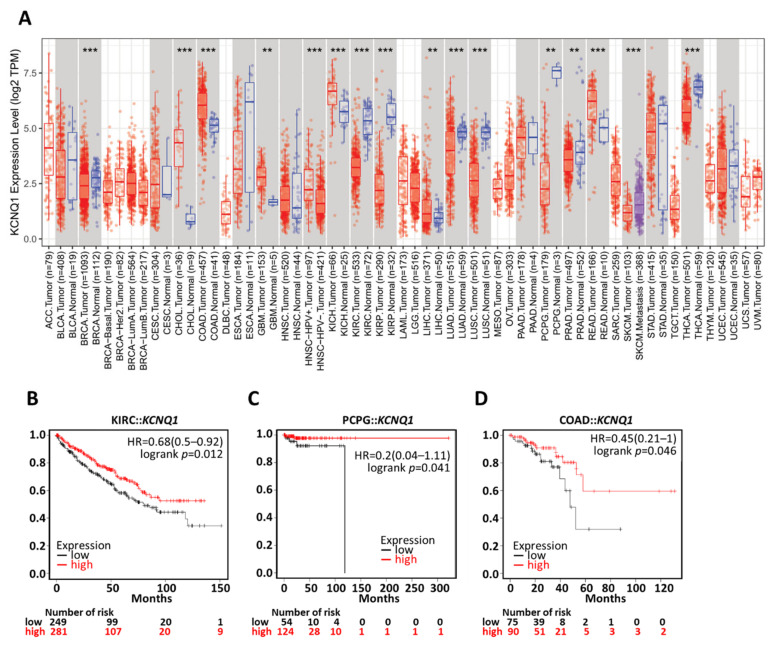
Generalization value of KCNQ1 in pan-cancer diagnosis and prognosis. Expression profiles of KCNQ1 by TNM plotter analysis (**A**). Kaplan–Meier survival analysis representing the probability of overall survival based on low/high KCNQ1 expression in kidney renal clear cell carcinoma (KIRC) (**B**), pheochromocytoma and paraganglioma (PCPG) (**C**), and colorectal adenocarcinoma (COAD) (**D**). ** *p* < 0.01, *** *p* < 0.001 tumor vs. normal.

To verify whether *KCNQ1* has a broad value, we investigated differential expression across cancers and confirmed the prognostic value by means of a Kaplan–Meier analysis. We noted that a number of cancers presented similar expression patterns to LUAD, such as breast cancer (BRCA), kidney renal clear cell carcinoma (KIRC), kidney renal papillary cell carcinoma (KIRP), lung squamous cell carcinoma (LUSC), pheochromocytoma and paraganglioma (PCPG), and thyroid carcinoma (THCA) (Figure 9A). The Kaplan–Meier analysis demonstrated that high *KCNQ1* expression had significant associations with longer overall survival time in KIRC (Figure 9B), PCPG (Figure 9C), and COAD (Figure 9D).

### 2.8. Lapatinib as a Therapeutic Option in the Context of Low KCNQ1

**Figure 10:**
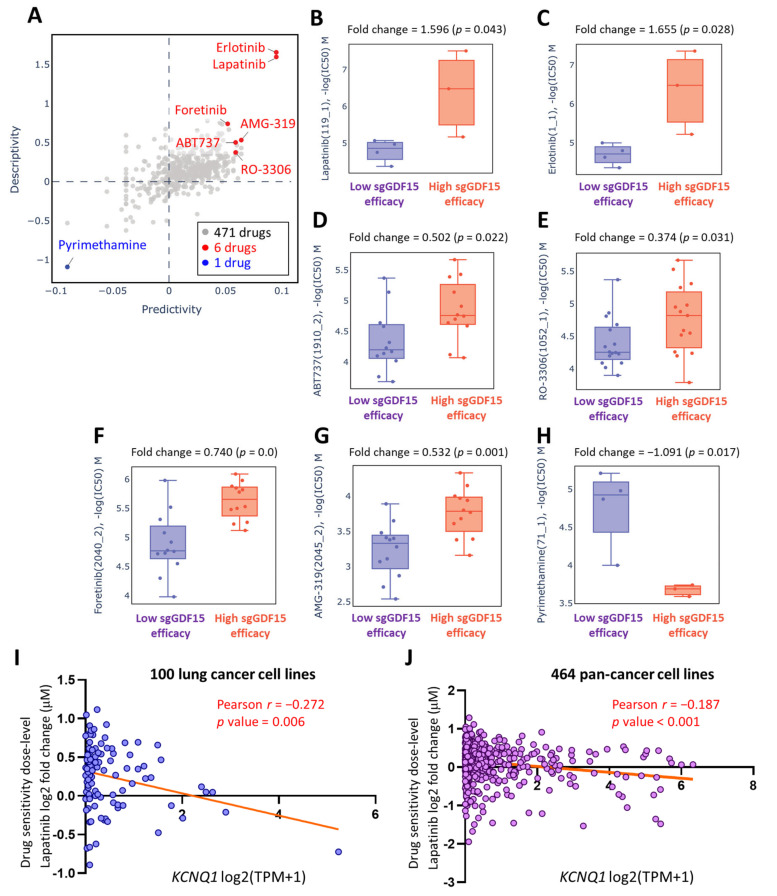
Lapatinib as a therapeutic option in the context of low *KCNQ1*. A Q-omics analysis was used to analyze cross-association scores regarding predictivity and descriptivity for the identification of potent drugs acting on LUAD cells based on the expression of *KCNQ1*. Predictivity denotes the fold change of single guide *KCNQ1* (sgKCNQ1) efficacy (sgRNA efficiency of *KCNQ1* knockout) between cells of high and low response to the target drug. Descriptivity denotes the fold change of target drug response between samples of high and low sgKCNQ1 efficiency. Red and blue dots represent hits with a predictivity *p*-value of < 0.05 and a descriptivity *p*-value of < 0.05 (**A**). Boxplots of −log(half maximal inhibitory concentration (IC50)) M of lapatinib (**B**), erlotinib (**C**), ABT737 (**D**), RO-3306 (**E**), foretinib (**F**), AMG-319 (**G**), and pyrimethamine (**H**). The relationship between *KCNQ1* expression levels and lapatinib sensitivity in 100 lung cancer cell lines (**I**) and 464 pan-cancer cell lines (**J**). The Pearson’s correlation of *KCNQ1* expression levels were expressed as the log2 of transcripts per million (TPM) and lapatinib sensitivity was expressed as the log of fold change (μM) to the base of 2.

With a view to exploring possible pharmaceutical approaches that could effectively target LUAD, we utilized the Genomics of Drug Sensitivity in Cancer (GDSC) repository of Q-omics analyses to identify drugs that feature potentiation effects in the context of low *KCNQ1* expression. We conducted cross-associations between drug response and single-guide RNA (sgRNA)-mediated knockdown of *KCNQ1* using a CRISPR approach in LUAD cell groups. Among 471 drugs, 7 drugs exerting altered potency were identified (Figure 10A and Supplementary File S4). LUAD cells with high sgRNA-GDF15 efficiency exhibited higher log(IC50) values in response to lapatinib (Figure 10B), erlotinib (Figure 10C), ABT737 (Figure 10D), RO-3306 (Figure 10E), foretinib (Figure 10F), and AMG-319 (Figure 10G). In contrast, pyrimethamine showed an inverse effect (Figure 10H). Furthermore, we employed a Cancer Cell Line Encyclopedia (CCLE) analysis to further verify the effect of *KCNQ1* expression levels on drug sensitivity in cancer cell lines. The drug sensitivity of lapatinib showed a negative correlation with KCNQ1 expression in 100 lung cancer cell lines (*r* = −0.272, *p* value = 0.006) (Figure 10I and Supplementary File S5) as well as 464 pan-cancer cell lines (*r* = −0.187, *p* value < 0.001) (Figure 10J and Supplementary File S6).

## 3. Discussion

**Figure 11:**
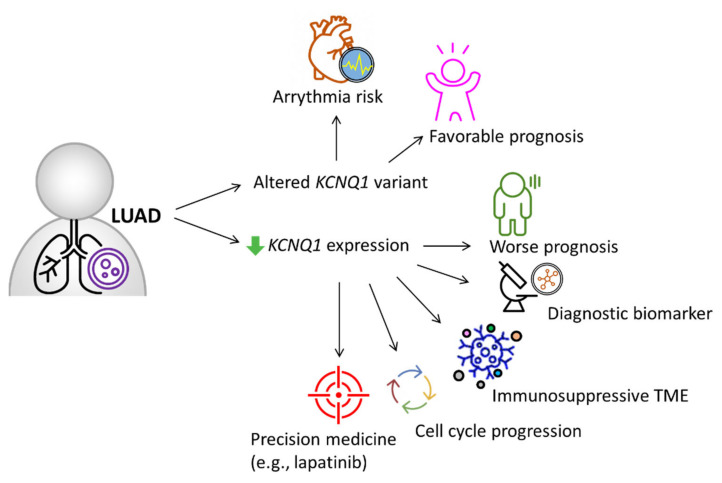
Proposed model illustrating the significance of *KCNQ1* in diagnosis, prognosis, immunity of the tumor microenvironment, tumor cell cycle, and precision treatment with gemcitabine in LUAD, aside from its recognized role in arrythmia risk [26].

*KCNQ1* is curated as one of the definitive genes for arrythmia [26], while its clinical implications and biological roles in LUAD remain unclear. In this study, we integrated multi-omics databases and experimental investigation to reveal *KCNQ1* as an independent risk predictor and as a diagnostic biomarker for LUAD. *KCNQ1* with altered variants had associations with favorable prognosis, while its low expression predicted worse prognosis and negatively correlated with tumor proliferation indicator Ki67. The *KCNQ1* mutations were highly associated with TMB. The decreased gene/protein expression of KCNQ1 can serve as a diagnostic biomarker and show good diagnostic efficiency. The *KCNQ1* expression preferentially showed prognostic value in the early stage of LUAD. On the other hand, we uncovered that the content of Th1, Th2, macrophages, NKT, MSC, and TMB can counteract the prognostic significance. Low *KCNQ1* is closely associated with decreased immune infiltration of CD8+ T cells, B cells, and M2 macrophages, which indicated shorter overall survival among the LUAD cohort. Specifically, *KCNQ1* expression negatively correlated with the levels of immunosuppressive molecules, wherein methylation modification may play a role. The potential mechanism of *KCNQ1* underlying LUAD progression may lie in the perturbation of genes relevant to the cell cycle and DNA replication. In addition, we showed that the diagnostic and prognostic value of *KCNQ1* can be extrapolated to other cancer types, including KIRC, PCPG, and COAD. Importantly, we identified that lapatinib can act as a therapeutic option when LUAD presents with low *KCNQ1* expression, offering a promising precision treatment strategy. A proposed model is summarized in Figure 11.

Recently, several studies have shown the clinical implication of *KCNQ1* in gastrointestinal cancers. Than et al. demonstrated that low expression of *KCNQ1* was associated with poor overall survival in patients with colorectal cancer [27]. den Uil et al. reported that low expression of KCNQ1 was associated with poor disease-free survival [28]. Yang et al. demonstrated that the KCNQ1 rs231348 CT variant indicates an increased gastric cancer risk [29]. Similar to the aforementioned observations, our study demonstrated that altered variants and high expression of *KCNQ1* indicated a favorable outcome in patients with LUAD and that its low expression represented a diagnostic biomarker of LUAD tumors.

Our study revealed that high *KCNQ1* expression intimately correlated with an immune infiltration profile that is associated with longer survival time and with downregulation of immunomodulatory genes, which may concurrently act to shape an immunoactive tumor microenvironment. In this regard, Than et al. demonstrated that *KCNQ1* loss-of-function in mice led to increased intestinal tumors and dysregulation of genes involved in immune homeostasis [27], in support of our observation.

In terms of the underlying mechanism of *KCNQ1* in tumor biology, Rapetti-Mauss et al. reported that *KCNQ1* acted to reduce proliferation and invasion of colorectal cancer cells by inhibiting the Wnt/β-catenin pathway [30]. Huang et al. demonstrated that overexpression of KCNQ1 decreased tumor growth and lung metastasis in a nude mouse model of renal cell carcinoma via a miR-140-5p/KLF9/KCNQ1 pathway [20]. In addition, Chen et al. noted that miR-483-5p acted to target KCNQ1, leading to facilitated cell proliferation and the invasion of esophageal cancer cells [19]. In line with these observations, we identified *KCNQ1* as an independent risk factor and a tumor suppressor in LUAD. We explored whether KCNQ1 may exert an inhibitory effect on pathways involved in the cell cycle and DNA replication. Specifically, the inhibition of DBF4, CCNA2, CCNB1, MCM6, CDC45, CHEK1, and CDC6 may be implicated in KCNQ1-mediated tumor suppression. Nevertheless, further investigation is warranted to elucidate the molecular basis.

Given that KCNQ1 plays a tumor suppressive role, its downregulation may promote the proliferation and metastasis of cancer cells. ShRNA-based knockdown of KCNQ1 was shown to promote cytosolic accumulation of β-catenin, which acts to mediate the proliferation of cancer cells [30]. Similar to our study, we noted that *KCNQ1* knockdown increased the migration ability of lung cancer cells. It is noteworthy that *KCNQ1* can be inhibited by gefitinib, which is the first-generation targeted therapy for NSCLC [31]. This KCNQ1-inhibiting effect of gefitinib is implicated in the induction of heart QT prolongation in a guinea pig model, thereby raising a concern of arrythmia when gefitinib is used for NSCLC treatment. Of note, the present study unveiled that lapatinib may be a potential therapeutic drug for the treatment of lung cancer when *KCNQ1* expression levels are low. While more studies are warranted, this finding may support the development of a precision treatment for LUAD based on *KCNQ1* expression levels.

## 4. Materials and Methods

### 4.1. Multi-Omics Analysis of KCNQ1 in Human Diseases and Cancers

The functional involvement of *KCNQ1* in human diseases was examined using the Open Target Platform (https://www.opentargets.org/; accessed on 12 January 2022), which integrates public data relevant to the association between targets and diseases and provides additional data and tools for prioritization. The relationships between *KCNQ1* variants and overall survival, disease-specific survival, progression-free survival and the mutational landscape of *KCNQ1* were retrieved from the cBioPortal for Cancer Genomics (https://www.cbioportal.org/; accessed on 12 January 2022) [32], which is a web platform of gene-based data exploration.

The Pearson correlation coefficient analysis for *KCNQ1* expression and its coexpression genes was analyzed using LinkedOmics [25], which is a multi-omics web portal for the analysis of 32 cancer types (http://www.linkedomics.org/login.php; accessed on 9 January 2022), presenting the results with volcano plots and heatmaps. The functional modules of Linkedomics were based on the Hiseq RNA platform. The gene set enrichment analysis (GSEA) was performed for and used in the analysis of biological process terms of gene ontology (GO) and Kyoto Encyclopedia of Genes and Genomes (KEGG) pathways.

For the protein–protein interaction (PPI) network analysis, we selected *KCNQ1* and the GSEA core enrichment genes and inputted them into GeneMANIA, which is an open website for building PPI networks and demonstrating gene function and data regarding physical interaction, co-expression, and co-location, as well as enrichment and predictive analyses [33,34].

### 4.2. Cell Culture and Transfection

Human lung adenocarcinoma epithelial cell lines A549 purchased from American Type Tissue Collection (ATCC, Manassas, VA, USA) were cultured in DMEM medium supplemented with 10% fetal bovine serum (FBS) (Thermo Fisher Scientific, Waltham, MA, USA) and 1% penicillin/streptomycin (Thermo Fisher Scientific, Waltham, MA, USA). Then, 20 nM of FAM-labeled negative control siRNA and *KCNQ1*-targeting siRNA (Thermo Fisher Scientific, Waltham, MA, USA) were transfected with Lipofectamine^TM^ RNAiMAX Transfection Reagent (Invitrogen, Carlsbad, CA, USA). siRNA transfection efficiency was monitored under a fluorescence microscope.

### 4.3. Wound Healing Assay

To allow attachment, 70 μL of the detached cells (2 × 10^5^ cells/mL) were seeded (ibidi culture-insert 2 well, ibidi GmbH, Martinsried, Germany) overnight. The culture insert was then removed, resulting in a bar of wound. Phosphate buffer solution was used to gently wash out the floating cells. The culture plate was photographed to document the width of the wound under a light microscope. The area of the wound was quantified using ImageJ Version 1.53i.

### 4.4. Gene/Protein Differential Expression and Prognostic Significance

The *KCNQ1* expression levels in LUAD tumors and adjacent normal tissue were accessed in TNMplot (https://tnmplot.com/analysis/; GEO datasets accessed on 12 January 2022) [35], in UALCAN (http://ualcan.path.uab.edu/; TCGA datasets accessed on 11 January 2022) [36,37], and in the Gene Expression Omnibus (GEO) (https://www.ncbi.nlm.nih.gov/geo/; GSE11502 datasets accessed on 10 January 2022). The immunohistochemical (IHC) staining signal of KCNQ1 protein expression was analyzed using the Human Protein Atlas (http://www.proteinatlas.org; accessed on 15 January 2022) [38,39,40]. The diagnostic efficiency was evaluated by means of the receiver operating characteristics (ROC) curve analysis. The survival analysis of various clinical stages and in the context of different immune cell contents or tumor mutational burdens was conducted using the Kaplan–Meier plotter web server (available online: https://kmplot.com/analysis/; accessed on 25 October 2021). The pan-cancer profile of *KCNQ1* expression was analyzed using the TNM plotter (https://tnmplot.com/analysis/; GEO datasets accessed on 12 January 2022).

### 4.5. Analysis of Immune Infiltration and Immunomodulator Profiling

The matrix content (stromal score), immune cell infiltration levels (immune score), and comprehensive score (ESTIMATE score) of TCGA-LUAD samples were evaluated using ESTIMATE [24]. The subtypes of tumor-infiltrating immune cells were determined using the TIMER online database [41]. The association between immune cell contents (CD8+ T cells, B cells, and M2 macrophages) and overall survival of LUAD patients was examined with Q-omics v.0.95 (accessed on 12 January 2022) [42]. The Spearman correlation test for *KCNQ1* expression and analysis of immunomodulator expression and methylation levels was conducted with TISIDB, which is a web portal for the analysis of tumor and immune system interactions [43].

### 4.6. Exploration of Potent Drugs Based on KCNQ1 Expression

The drug sensitivity profiling based on *KCNQ1* expression was analyzed using the CRISPR-screen data repository of the GDSC algorithm in Q-omics v.1.0 (accessed on 12 January 2022) [42]. The data of cellular sensitivity to lapatinib based on KCNQ1 expression was retrieved from the Cancer Cell Line Encyclopedia (CCLE) (https://sites.broadinstitute.org/ccle/tools; accessed on 18 January 2022) [44]. The Pearson correlation coefficient analysis was used to analyze the correlation between KCNQ1 expression levels and lapatinib dose levels.

### 4.7. Statistical Analysis

An unpaired *t*-test was used to compare quantitative data for two groups. The Pearson method was employed to analyze correlations. The log-rank method was used to test the survival difference between the two groups. GraphPad Prism V8.0 (GraphPad Software, San Diego, CA, USA) and SPSS V18.0 (IBM, Armonk, NY, USA) were used for statistical processing. GraphPad Prism V8.0 was utilized for data visualization.

## 5. Conclusions

This study unveiled the significance of *KCNQ1* in diagnosis/prognosis and provided a corresponding precision medicine strategy.

## Data Availability

Data are contained within the article.

## Supplementary Materials

The following supporting information can be downloaded at: www.mdpi.com/article/10.3390/ijms23042279/s1.
